# Cognitive and Behavioral Development of 9-Year-Old Children After Maternal Cancer During Pregnancy: A Prospective Multicenter Cohort Study

**DOI:** 10.1200/JCO.22.02005

**Published:** 2023-01-12

**Authors:** Indra A. Van Assche, Evangeline A. Huis in 't Veld, Kristel Van Calsteren, Mathilde van Gerwen, Jeroen Blommaert, Elyce Cardonick, Michael J. Halaska, Robert Fruscio, Monica Fumagalli, Jurgen Lemiere, Elisabeth M. van Dijk-Lokkart, Camilla Fontana, Harm van Tinteren, Jessie De Ridder, Martine van Grotel, Marry M. van den Heuvel-Eibrink, Lieven Lagae, Frédéric Amant

**Affiliations:** ^1^ Department of Development and Regeneration, Unit of Woman and Child, KU Leuven, Belgium; ^2^ Princess Máxima Center for Pediatric Oncology, Utrecht, the Netherlands; ^3^ Center for Gynecological Oncology, Netherlands Cancer Institute, Amsterdam, the Netherlands; ^4^ Division of Foetomaternal Medicine, Department of Obstetrics and Gynaecology, UZ Leuven, Belgium; ^5^ Department of Child and Adolescent Psychiatry and Psychosocial Care, Amsterdam UMC, University of Amsterdam, the Netherlands; ^6^ Department of Oncology, Unit of Pediatric Oncology, KU Leuven, Belgium; ^7^ Department of Oncology, Unit of Gynaecological Oncology, KU Leuven, Belgium; ^8^ Department of Oncology, Laboratory of Experimental Radiotherapy, KU Leuven, Belgium; ^9^ Department of Obstetrics and Gynecology, Cooper University Health Care, Camden, NJ; ^10^ Department of Obstetric Gynecology, University Hospital Kralovske Vinohrady and 3rd Medical Faculty, Charles University, Prague, Czech Republic; ^11^ Department of Medicine and Surgery, Clinic of Obstetrics and Gynecology, University of Milan-Bicocca, San Gerardo Hospital, Monza, Italy; ^12^ Department of Clinical Sciences and Community Health, University of Milan, Milano, Italy; ^13^ Fondazione IRCCS Ca' Granda Ospedale Maggiore Policlinico, NICU, Milano, Italy; ^14^ Division of Pediatric Hemato-Oncology, Department of Pediatrics, UZ Leuven, Belgium; ^15^ Amsterdam Reproduction and Development, Child Development, Amsterdam, the Netherlands; ^16^ Division of Pediatric Neurology, Department of Pediatrics, UZ Leuven, Belgium; ^17^ Department of Development and Regeneration, Unit Locomotor and Neurological Disorders, KU Leuven, Belgium; ^18^ UMCU-Wilhelmina Children's Hospital, Utrecht, the Netherlands; ^19^ Division of Gynaecological Oncology, Department of Obstetrics and Gynaecology, UZ Leuven, Belgium

## Abstract

*Clinical trials frequently include multiple end points that mature at different times. The initial report, typically based on the primary end point, may be published when key planned co-primary or secondary analyses are not yet available. Clinical Trial Updates provide an opportunity to disseminate additional results from studies, published in* JCO *or elsewhere, for which the primary end point has already been reported.*

This multicenter cohort study reports on the long-term effects of prenatal exposure to maternal cancer and its treatment on cognitive and behavioral outcomes in 9-year-old children. In total, 151 children (mean age, 9.3 years; range, 7.8-10.6 years) were assessed using a neurocognitive test battery and parent-report behavioral questionnaires. During pregnancy, 109 children (72.2%) were exposed to chemotherapy (only or in combination with other treatment modalities), 18 (11.9%) to surgery only, 16 (10.6%) to radiotherapy, one to trastuzumab, and 16 (10.6%) were not exposed to oncologic treatment. Mean cognitive and behavioral outcomes were within normal ranges. Gestational age at birth showed a positive association with Full Scale Intelligence Quotient (FSIQ), with the average FSIQ score increasing by 1.6 points for each week increase in gestational age (95% CI, 0.7 to 2.5; *P* < .001). No difference in FSIQ was found between treatment types (F[4,140] = 0.45, *P* = .776). In children prenatally exposed to chemotherapy, no associations were found between FSIQ and chemotherapeutic agent, exposure level, or timing during pregnancy. These results indicate a reassuring follow-up during the critical maturational period of late childhood, when complex functions develop and rely on the integrity of early brain development. However, associations were observed with preterm birth, maternal death, and maternal education.

## INTRODUCTION

Maternal cancer during pregnancy is an emerging challenge and may affect child development through both direct treatment effects and indirect environmental and psychosocial effects, in both prenatal and postnatal periods.^1^ As part of the International Network on Cancer, Infertility and Pregnancy, our group previously published reports showing reassuring general outcomes of children prenatally exposed to maternal cancer and its treatment.^2-7^ Nevertheless, specific results highlight a need for continued follow-up. At a median age of 22 months, prematurity predicted poorer cognitive outcomes.^2,3^ At age 6 years, prenatally exposed children showed lower verbal intelligence and visuospatial long-term memory scores, and chemotherapy during pregnancy was associated with poorer emotional regulation.^6,7^ Maternal death also represented an important risk factor for poorer neurocognitive and behavioral outcomes.^7-10^

The question remains whether children born after a pregnancy complicated by maternal cancer are at risk of growing into deficits when complex cognitive and executive functions are developing and relying on brain structures that developed aberrantly during gestation and early childhood.^11,12^ This report describes the cognitive and behavioral outcomes of 9-year-old children prenatally exposed to maternal cancer and its treatment.

## METHODS

### Study Design

The INCIP Child Follow-up study (Protocol, online only) evaluates the general health and neurocognitive development after prenatal exposure to maternal cancer (treatment) using age-adapted standardized test batteries.^7^ Children are included longitudinally at the ages 18 months, 36 months, and subsequently once every 3 years until age 18 years.

We report a cross-sectional analysis of the 9-year-old children (Data Supplement, online only). Ethical approval and written informed consent was obtained for all participating subjects.

### Outcomes

A neuropsychologic examination, assessing intelligence quotient, attention, memory, and behavior, was performed (Data Supplement). The primary outcome was the Full Scale Intelligence Quotient (FSIQ), derived from the Wechsler Intelligence Scale for Children.^13-15^ Secondary outcomes included all other neurocognitive test scores and behavioral questionnaire results. All children underwent a clinical neurologic and general pediatric examination, and parents completed a questionnaire on their child's general health and educational level. All tests were conducted in the child's native language.

Methodologic details are reported in the Data Supplement.

## RESULTS

### Participants

A total of 151 children (including seven pairs of twins) were included: 95 from Belgium, 34 from Netherlands, nine from Italy, six from Czech Republic, and seven from New Jersey (Table 1). The Data Supplement contains further information about maternal cancer types, treatment characteristics, substance use during pregnancy, fertility treatment, bilingualism, congenital malformations, and labor types and delivery modes.

**TABLE 1.**
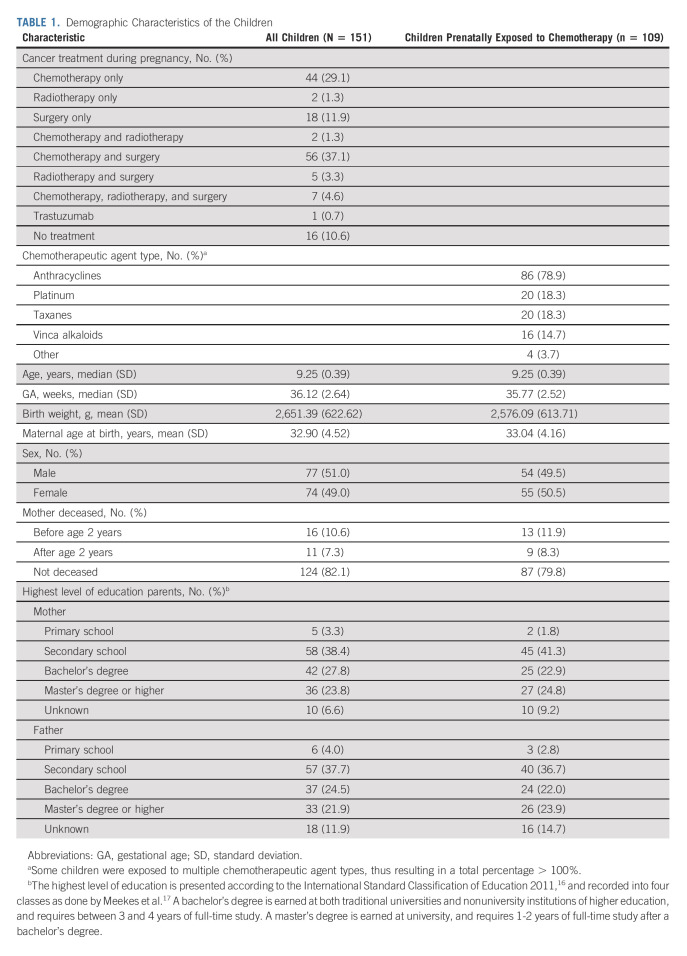
Demographic Characteristics of the Children

### Cognitive Development and Behavior

**FIG 1.**
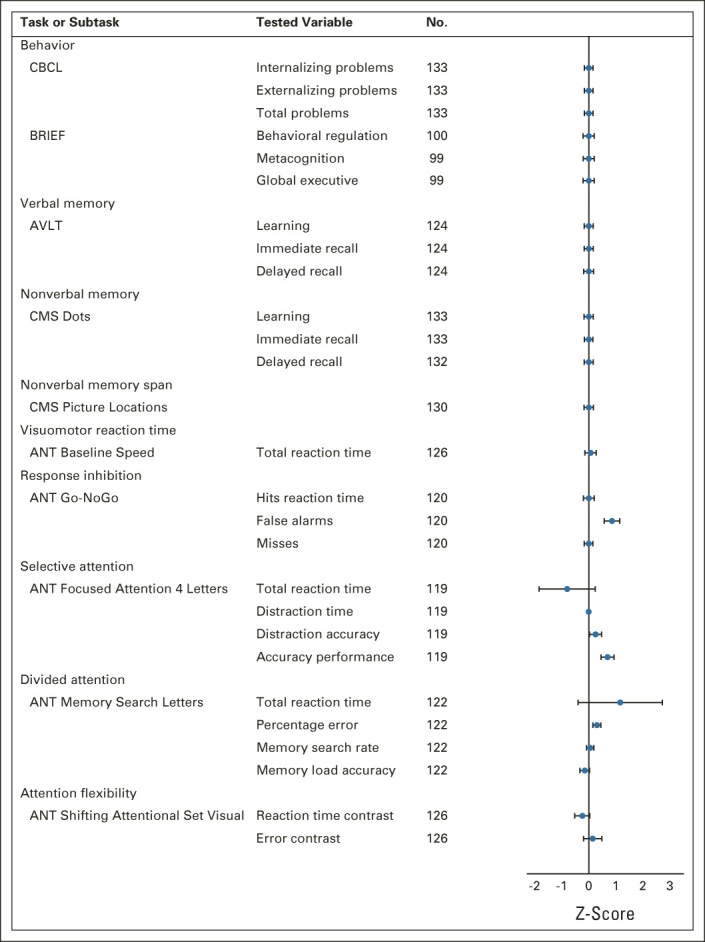
Cognitive (nonintelligence quotient) and behavioral outcomes. ANT, Amsterdam Neuropsychologic Task; AVLT, Auditory Verbal Learning Test; BRIEF, Behavior Rating Inventory of Executive Function; CBCL, Child Behavior Checklist; CMS, Children’s Memory Scale.

Group outcomes for all intelligence outcomes, verbal and visuospatial memory, attentional function, and behavioral measures were within normal ranges (Table 2 and Fig 1).

**TABLE 2.**
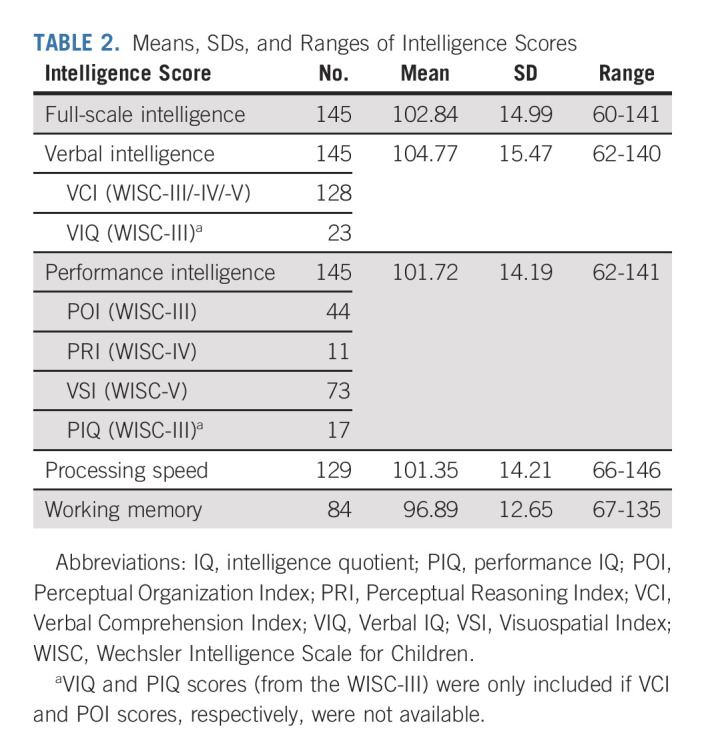
Means, SDs, and Ranges of Intelligence Scores

No difference in FSIQ was found between girls and boys (t[143] = 0.25, *P* = .802), treatment types (F[4,140] = 0.45, *P* = .776), or cancer stages (F[3,140] = 1.53, *P* = .211).

Children who scored below normal ranges were more likely to have been born preterm. The average FSIQ score increased by 1.6 points (95% CI, 0.7 to 2.5; *P* < .001; Data Supplement) for each week increase in gestational age at birth (GA). GA also explained verbal intelligence (β = 1.49 points/wk; CI, 0.6 to 2.4; *P* = .002), performance intelligence (β = 1.39 points/wk; CI, 0.6 to 2.2; *P* = .002), and processing speed (β = 1.21 points/wk; CI, 0.3 to 2.1; *P* = .009).

We found an effect of maternal bereavement (F[2, 144] = 3.94, *P* = .022). FSIQ was lower in children with a deceased mother before age 2 years (93.13 ± 12.65) than in children with a surviving mother (104.08 ± 14.88). When adjusting for GA, this association disappeared (*P* > .3): Children with a deceased mother before age 2 years were on average born earlier (33.4 weeks ± 2.7) than children with a deceased mother after age 2 years (37.1 weeks ± 1.7) and children with a surviving mother (36.4 weeks ± 2.5; *P* < .001). No associations were found between maternal death and cancer type, cancer stage, or treatment type.

Multiple linear regression (Data Supplement) with GA, death of mother, and parental (maternal and paternal) education level as explanatory variables shows that GA (*P* = .006) and maternal education level (*P* = .005) remained to explain FSIQ. Specifically, this model estimated an increase of 1.27 points (± 0.45) in FSIQ for every week increase in GA.

When looking specifically at the subgroup of children prenatally exposed to chemotherapy, a mixed-effect regression model, with parental education levels as random variables, found no significant associations with FSIQ (all *P* > .08). When parental education levels were separately included as fixed effects, maternal education level remained a significant predictor of FSIQ (*P* = .011).

### General Health

Data from the parent-reported health questionnaire revealed no specific problems across the group. An overview of the reported medical problems is enlisted in the Data Supplement.

Four children were diagnosed with attention-deficit hyperactivity disorder, of which three took supportive medication. One child was diagnosed with autism spectrum disorder. Thirty-one children (24.4%) received remedial care, including speech therapy (17 children), remedial teaching at school (10), a type of physical or exercise therapy (four), and neurofeedback therapy (one).

From these children receiving remedial support, eight children (25.8%) lost their mother (of which seven before age 2 years), with a chi-squared test showing a significant relation between maternal bereavement before age 2 years and remedial care (χ^2^ [2,127] = 7.33, *P* = .026, φ = 0.240). An association was also found between remedial support and prematurity: 24 children (77.4%) who received remedial care were born preterm (χ^2^ [1,127] = 6.67, *P* = .010, φ = 0.229).

## DISCUSSION

Cognitive and behavioral outcomes of 9-year-old children born to mothers diagnosed with cancer during pregnancy did not differ with norms of the general population. We reported average group scores for all intelligence index scores, verbal and visuospatial memory outcomes, attentional functioning outcomes, and behavioral questionnaire measures. We found no impact of sex, treatment type, and cancer stage.

Children who scored below normal ranges on intelligence index scores were more likely to have been born preterm. We report an increase in Full Scale Intelligence Quotient of almost 6.5 points for each month increase in GA. Hence, preterm birth should be avoided as much as possible in the obstetric management of pregnant women with cancer.

Seventeen percent of the children in this study had lost their mother (of which almost 58% died before their child turned 2 years old). The results suggest a relationship between maternal bereavement in the first 1,000 days of life and Full Scale Intelligence, although a larger sample is necessary to disentangle effects of maternal loss and often coinciding premature birth.

A longitudinal study in the general population also demonstrates multicollinearity between the impact of early-life adversity exposure and prematurity.^18^ As maternal death was not associated with cancer type, cancer stage, or treatment type, it could be possible that maternal stress or an insecure mother-child attachment may play a role in determining child cognitive development.

In the subgroup of children prenatally exposed to chemotherapy, only maternal education level was associated with Full Scale Intelligence. In this group, no effect of prematurity was found in both the current 9-year-old cohort and the 6-year-old cohort.^7^ Further research is needed to determine whether the effect of prematurity is hidden by an underpowered analysis in the smaller chemotherapy group, or whether administering chemotherapy during pregnancy perhaps reduces the beneficial effect of delivery at term.

Maternal education level explained part of the outcome on Full Scale Intelligence. This effect was found in the entire sample and chemotherapy subgroup. Although both paternal and maternal education level were associated with Full Scale Intelligence at the univariate level, only maternal education remained a significant predictor in fitted models.

Almost a quarter of parents reported a need for remedial support for their child. As rates of pediatric remedial support in the general population remain under-reported, disallowing a comparison of the observed rates and the rates of the general population, further research is needed to put this percentage in perspective. The need for remedial support may be related to prematurity and maternal bereavement, with 77% of the children who received support born preterm, and 26% of the children having lost their mother to cancer. In combination with a recent study showing increased child trauma–related cognitions and emotion regulation difficulties after parental cancer (bereavement),^19^ it tentatively supports the need to follow these families in the long term to determine their psychosocial needs.

In conclusion, children prenatally exposed to maternal cancer (treatment) showed on average a normal cognitive and behavioral development at age 9 years. No associations of cognition and behavior were found with treatment type, exposure level, cancer stage, and gestational age at the start of treatment. These results convey a reassuring follow-up of neurocognitive development during late childhood, which represents a time of critical maturation when complex functions are developing. However, these children still require a close follow-up, as maternal cancer during pregnancy is associated with preterm delivery and maternal death, which are risk factors for developmental problems.
